# Hydrogen Sulfide Releasing Aspirin, ACS14, Attenuates High Glucose-Induced Increased Methylglyoxal and Oxidative Stress in Cultured Vascular Smooth Muscle Cells

**DOI:** 10.1371/journal.pone.0097315

**Published:** 2014-06-04

**Authors:** Qian Huang, Anna Sparatore, Piero Del Soldato, Lingyun Wu, Kaushik Desai

**Affiliations:** 1 Department of Pharmacology, College of Medicine, University of Saskatchewan, Saskatoon, Saskatchewan, Canada; 2 King's Lab, School of Pharmacy, Shanghai Jiao Tong University, Shanghai, China; 3 Dipartimento di Scienze Farmaceutiche, Università degli Studi di Milano, Milan, Italy; 4 CTG Pharma, Milan, Italy; 5 Department of Health Sciences, Lakehead University, Thunder Bay, Ontario, Canada; 6 Thunder Bay Regional Research Institute, Thunder Bay, Ontario, Canada; Case Western Reserve University, United States of America

## Abstract

Hydrogen sulfide is a gasotransmitter with vasodilatory and anti-inflammatory properties. Aspirin is an irreversible cyclooxygenase inhibitor anti-inflammatory drug. ACS14 is a novel synthetic hydrogen sulfide releasing aspirin which inhibits cyclooxygenase and has antioxidant effects. Methylglyoxal is a chemically active metabolite of glucose and fructose, and a major precursor of advanced glycation end products formation. Methylglyoxal is harmful when produced in excess. Plasma methylglyoxal levels are significantly elevated in diabetic patients. Our aim was to investigate the effects of ACS14 on methylglyoxal levels in cultured rat aortic vascular smooth muscle cells. We used cultured rat aortic vascular smooth muscle cells for the study. Methylglyoxal was measured by HPLC after derivatization, and nitrite+nitrate with an assay kit. Western blotting was used to determine NADPH oxidase 4 (NOX4) and inducible nitric oxide synthase (iNOS) protein expression. Dicholorofluorescein assay was used to measure oxidative stress. ACS14 significantly attenuated elevation of intracellular methylglyoxal levels caused by incubating cultured vascular smooth muscle cells with methylglyoxal (30 µM) and high glucose (25 mM). ACS14, but not aspirin, caused a significant attenuation of increase in nitrite+nitrate levels caused by methylglyoxal or high glucose. ACS14, aspirin, and sodium hydrogen sulfide (NaHS, a hydrogen sulfide donor), all attenuated the increase in oxidative stress caused by methylglyoxal and high glucose in cultured cells. ACS14 prevented the increase in NOX4 expression caused by incubating the cultured VSMCs with MG (30 µM). ACS14, aspirin and NaHS attenuated the increase in iNOS expression caused by high glucose (25 mM). In conclusion, ACS14 has the novel ability to attenuate an increase in methylglyoxal levels which in turn can reduce oxidative stress, decrease the formation of advanced glycation end products and prevent many of the known deleterious effects of elevated methylglyoxal. Thus, ACS14 has the potential to be especially beneficial for diabetic patients pending further *in vivo* studies.

## Introduction

**Figure 1:**
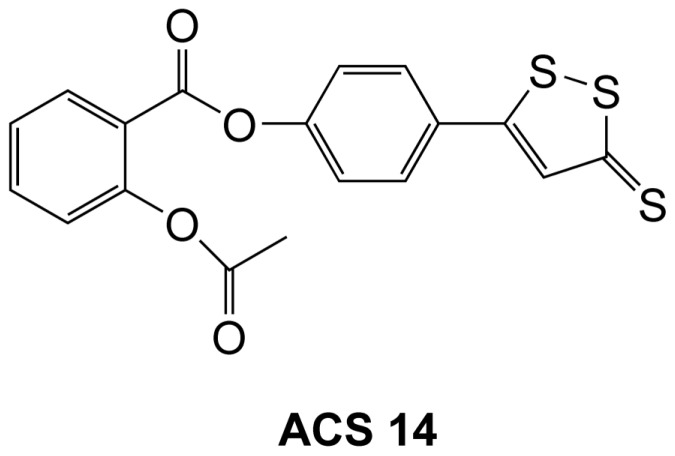
Chemical structure of H_2_S releasing aspirin, ACS14 [2-acetyloxybenzoic acid 4-(3-thioxo-3*H*-1,2-dithiol-5-yl)phenyl ester].

Hydrogen sulfide (H_2_S) is a physiological gasotransmitter molecule which has vasodilatory and anti-inflammatory properties [1], [2]. Aspirin (acetylsalicylic acid) is an irreversible cyclooxygenase inhibitor which is used as a non-steroidal anti-inflammatory drug. Low dose aspirin (81–160 mg/day) is widely used as an antiplatelet drug [3]. Aspirin has been shown to inhibit glycation of lens protein by acetylation [4]. ACS14 (2-acetyloxybenzoic acid 4-(3-thioxo-3*H*-1,2-dithiol-5-yl)phenyl ester, Fig. 1) is a novel synthetic H_2_S releasing aspirin which inhibits cyclooxygenase like aspirin, but it spares the gastric mucosa, by a favorable redox balance caused by increased H_2_S/glutathione (GSH) formation, heme oxygenase-1 (HO-1) expression and reduced 8-isoprostane-prostaglandin F_2α_ (8-isoprostane) formation [5]. ACS14 increases antioxidant defense by increasing GSH and cysteine and decreasing homocysteine levels [6].

Methylglyoxal (MG) (pyruvaldehyde) is produced mainly during glucose and fructose metabolism, and to a lesser extent during fatty acid and amino acid metabolism [7]. Chemically MG is a reactive dicarbonyl molecule which readily reacts with certain proteins and enzymes and disrupts their structure and function [8], [9]. MG is of great pathological significance because it is a major precursor for the formation of advanced glycation end products (AGEs) [10]. The glyoxalase enzymes and reduced glutathione (GSH) rapidly degrade physiological amounts of MG produced in the body into D-lactate [11], [12]. An excess of MG formation, as occurs in diabetic patients [13], causes a 3–4 fold elevation of plasma MG levels [14], [15], and is harmful.

We have shown that incubation of vascular smooth muscle cells (VSMCs) with 25 mM glucose or fructose for 3 h increases MG production 3.5 or 3.9 fold, respectively, and increases oxidative stress [16]. MG and high glucose also reduced nitric oxide (NO) production and caused endothelial dysfunction in cultured endothelial cells and isolated aortic rings [8]. Chronic treatment of Sprague-Dawley rats with MG for 4 weeks induces features characteristic of type 2 diabetes mellitus [17].

We have recently shown that H_2_S interacts with MG in cultured VSMCs, in which the H_2_S donor sodium hydrogen sulfide (NaHS, 30, 60 and 90 µM) significantly decreased cellular MG levels [18]. Therefore, our main aim was to see if ACS14 could prevent or attenuate the increase in intracellular MG levels and the associated oxidative stress, caused by high glucose or exogenous MG, and our results show that this is indeed the case.

## Methods

### Vascular smooth muscle cell culture

Rat thoracic aortic vascular smooth muscle cell line (VSMCs, A-10 cells, Cat # ATCC CRL-1476; American Type Culture Collection, Manassas, VA, USA) was cultured in Dulbecco's Modified Eagle's Medium (DMEM) containing 10% fetal bovine serum (FBS) at 37°C in a humidified atmosphere of 95% air and 5% CO_2_, as described previously [19]. A-10 cells were seeded either in 100 mm dishes for MG measurement or in 96-well plates for other assays, with an equal amount of cells (10^6^/ml) in each well, and cultured to confluence. Cells were starved in FBS-free DMEM for 24 h before exposure to different test reagents. The concentrations of MG and NaHS were determined from previous studies in our lab [16], [18].

### Methylglyoxal measurement

MG was measured by a specific and sensitive high-performance liquid chromatography (HPLC) method [20]. MG was derivatized with *o*-phenylenediamine (*o*-PD) to form the quinoxaline product, 2-methylquinoxaline, which is very specific for MG. For MG measurement the cells were washed twice with phosphate buffered saline (PBS), scrapped and cell pellets were resuspended in ice-cold PBS, and lysed over ice by sonication (5 s, three times). The samples were incubated in the dark for 24 h with 0.45 N perchloric acid and 10 mM *o*-PD at room temperature. The quinoxaline derivatives of MG (2-methylquinoxaline) and the quinoxaline internal standard (5-methylquinoxaline) were quantified on a Hitachi D-7000 HPLC system (Hitachi, Ltd., Mississauga, ON, Canada) *via* Nova-Pak C18 column (3.9×150 mm, and 4 µm particle diameter, Waters Corporation, MA, USA).

### Measurement of nitrite and nitrate

Cells were incubated with different test reagents for 24 h and then washed with PBS. The supernatant was used for the measurement of nitrite and nitrate with a fluorimetric assay kit (Cat # 780051, Cayman Chemical Company, Ann Arbor, MI, USA) based on the Greiss reaction. The assay is based on the enzymatic conversion of nitrate to nitrite by nitrate reductase followed by the addition of 2,3-diaminonaphthalene, which converts nitrite to a fluorescent compound. Fluorescence intensity measurements of this compound accurately determine the nitrite (NO_2_) concentration (excitation max.: 365 nm; emission max.: 450 nm).

### Measurement of oxidative stress

Oxidative stress was determined by a sensitive dicholorofluorescein (DCFH) assay. Briefly, cells were loaded with a membrane-permeable, nonfluorescent probe 2′, 7′-dichlorofluorescein diacetate (CM-H_2_DCFDA, 5 µM) for 2 h at 37°C in FBS-free DMEM in the dark. After washing 3 times with PBS, the cells were treated with or without different substrates or MG for different incubation times, and finally subjected to detection. Once inside the cells, CM-H_2_DCFDA becomes membrane-impermeable DCFH_2_ in the presence of cytosolic esterases, and is further oxidized by peroxynitrite to form the fluorescent oxidized dichlorofluorescein (DCF). The probe has high reactivity with peroxynitrite and its products CO_3_ ˙^−^ and ˙NO_2_ but is not entirely specific for it. It also has low reactivity for hydrogen peroxide and even lower for superoxide [21]. The fluorescence intensity was measured with excitation at 485 nm and emission at 527 nm utilizing a Fluoroskan Ascent plate reader (Thermo Labsystems, Fisher Scientific Co., Ottawa, ON, Canada) and Ascent software, and expressed in arbitrary units.

### Western blotting

Cell lysate was separated by 8% or 10% SDS-PAGE, electrotransferred onto a polyvinylidene fluoride membrane, blocked with 5% skim milk for 30 minutes and incubated with primary antibodies diluted in skim milk overnight at 4°C. The next day, after 2 h of thorough washing with PBST buffer (PBS with 0.1% tween-20), the membranes were incubated with horseradish peroxidase-conjugated secondary antibodies for 2 h at room temperature. After 1 h washing, the immunoreactive proteins were detected with an Enhanced Chemiluminescence Detection System. Primary antibody for NADPH oxidase 4 (NOX4) was purchased from Santa Cruz (Santa Cruz Biotechnology Inc., Santa Cruz, CA, USA). iNOS antibody was from BD Transduction Laboratories (BD Biosciences, Mississauga, ON, Canada). β-actin was purchased from Sigma (Sigma-Aldrich Corp., St. Louis, MO, USA), and secondary anti-rabbit and anti-mouse IgG antibodies were from Cell Signaling (Cell Signaling Technology Inc., Danvers, MA, USA).

### Cell viability assay

Cell viability was determined with a CellTiter 96 AQueous One Solution Cell Proliferation Assay with a kit from Promega (Promega Corp., Madison, WI, USA), following the manufacturer's instructions. The assay uses MTS tetrazolium compound [3-(4,5-dimethylthiazol-2-yl)-5-(3-carboxymethoxyphenyl)-2-(4-sulfophenyl)-2H-tetrazolium, inner salt] and phenazine ethosulfate (PES), an electron coupling reagent. MTS is converted into a soluble formazan product by living cells. The amount of formazan produced correlates with viable cells. Briefly, VSMCs (A-10 cells, 10^5^ cells/well) were plated into 96-well tissue culture plates. After incubation with MG (30 µM) or ACS14 (30, 100 or 300 µM) alone or in combination in 100 µl of FBS-free DMEM at 37°C for 24 h, 20 µl of CellTiter 96 AQueous One Solution Reagent was added to each well. After a further incubation for 4 h at 37°C in a 5% CO_2_ atmosphere, absorbance was measured at 490 nm using a Multiskan Spectrum plate reader (Thermo Labsystems, Fisher Scientific Co., Ottawa, ON, Canada).

### Materials

ACS14 and aspirin were kindly provided by CTG Pharma, Milan, Italy. Methylglyoxal, D-glucose, aspirin and NaHS were purchased from Sigma-Aldrich Canada Ltd (Mississauga, ON, Canada). Chemical compounds: Chemical compounds studied in this article: 2-acetyloxybenzoic acid 4-(3-thioxo-3*H*-1,2-dithiol-5-yl)phenyl ester (ACS14); Aspirin (acetylsalicylic acid) (PubChem CID: 2244); Methylglyoxal (Pyruvaldehyde) (PubChem CID: 880); D-glucose (Dextrose) (PubChem CID: 5793); Sodium hydrogen sulfide (PubChem CID: 28015).

### Statistics

Statistical analysis was performed using one way ANOVA and Tukey's post-hoc test. *P*<0.05 was taken as significant.

## Results

### ACS14 significantly attenuates elevation of intracellular MG levels caused by MG and high glucose in cultured cells

**Figure 2:**
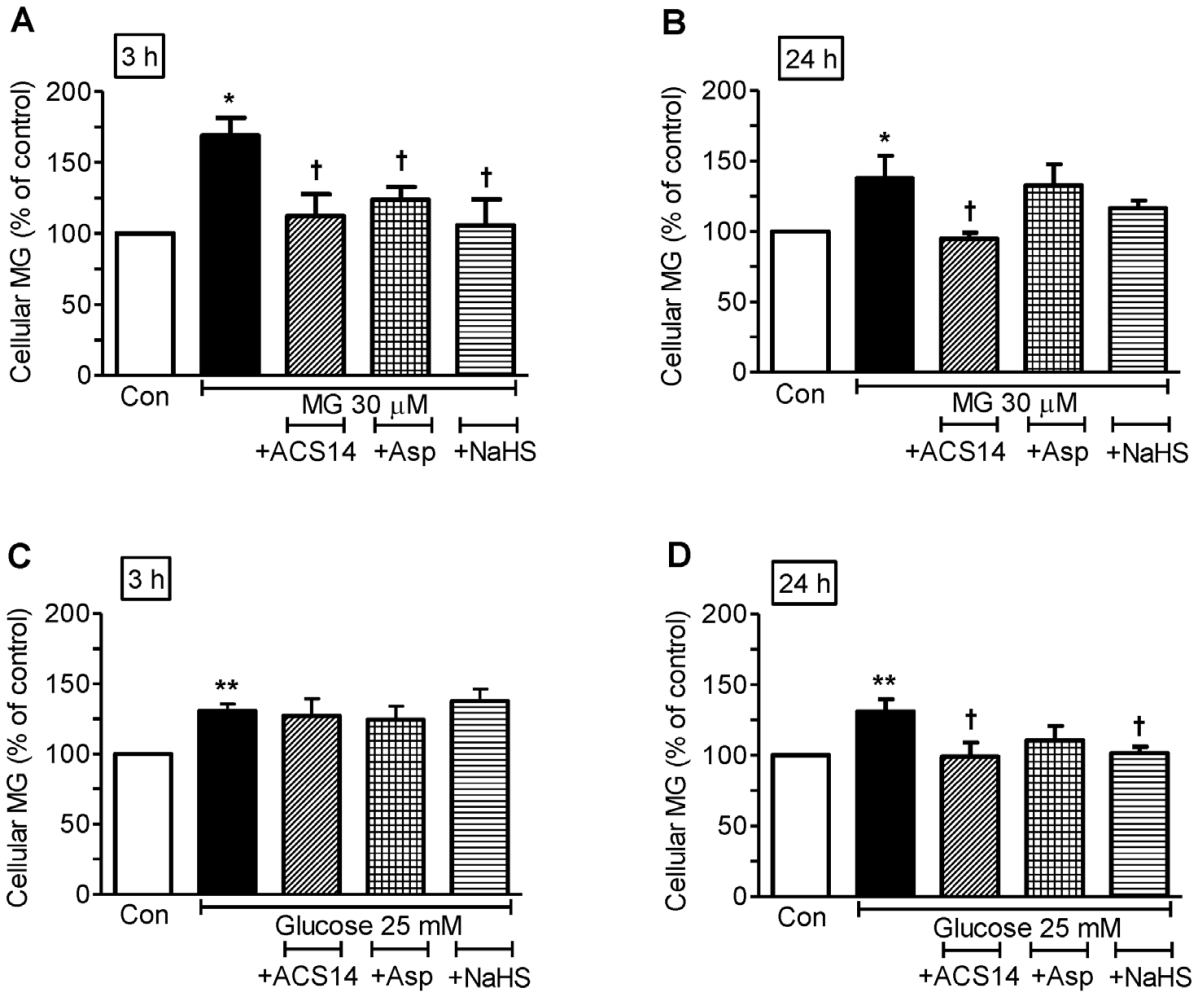
ACS14 significantly attenuates elevation of intracellular MG levels caused by MG and high glucose in cultured cells. Cultured rat aortic vascular smooth muscle cells (VSMCs, A10 cell line) were incubated with methylglyoxal (MG, 30 µM) or high glucose (25 mM) alone or co-incubated with either ACS14 (100 µM), or aspirin (100 µM) or sodium hydrogen sulfide (NaHS, 90 µM) for 3 h or 24 h. MG levels in the cells were measured after derivatizing MG with ortho-phenylenediamine to form 2-methylquinoxaline, which was detected with HPLC. *P<0.05 and **P<0.01 *vs.* respective control, ^†^ *P*<0.05 *vs.* respective MG group or high glucose group.

Incubation of cultured VSMCs with MG (30 µM) or high glucose (25 mM) for 3 or 24 h caused a significant elevation of intracellular MG levels (Fig. 2). Co-incubation with ACS14 significantly attenuated the increase in MG levels caused by 3 h or 24 h incubation with MG (Fig. 2A, B), or 24 h incubation with high glucose (Fig. 2D). Aspirin only significantly attenuated elevation of MG level caused by 3 h incubation with MG (Fig. 2A). NaHS caused a significant attenuation of increase in MG levels caused by 3 h incubation with MG and 24 h incubation with high glucose (Fig. 2A, D). The 3 h time point to measure MG levels was chosen based on our previous observation that MG levels in cultured VSMCs peaked at 3 h after incubation with fructose [22] and increased significantly at 3 h after incubation with glucose [16]. The 24 h time point was chosen as a standard time-point to measure changes in protein expression in cultured cells.

### ACS14, but not aspirin, causes a significant attenuation of increase in nitrate+nitrite levels and iNOS expression caused by MG and/or high glucose in cultured cells

**Figure 3:**
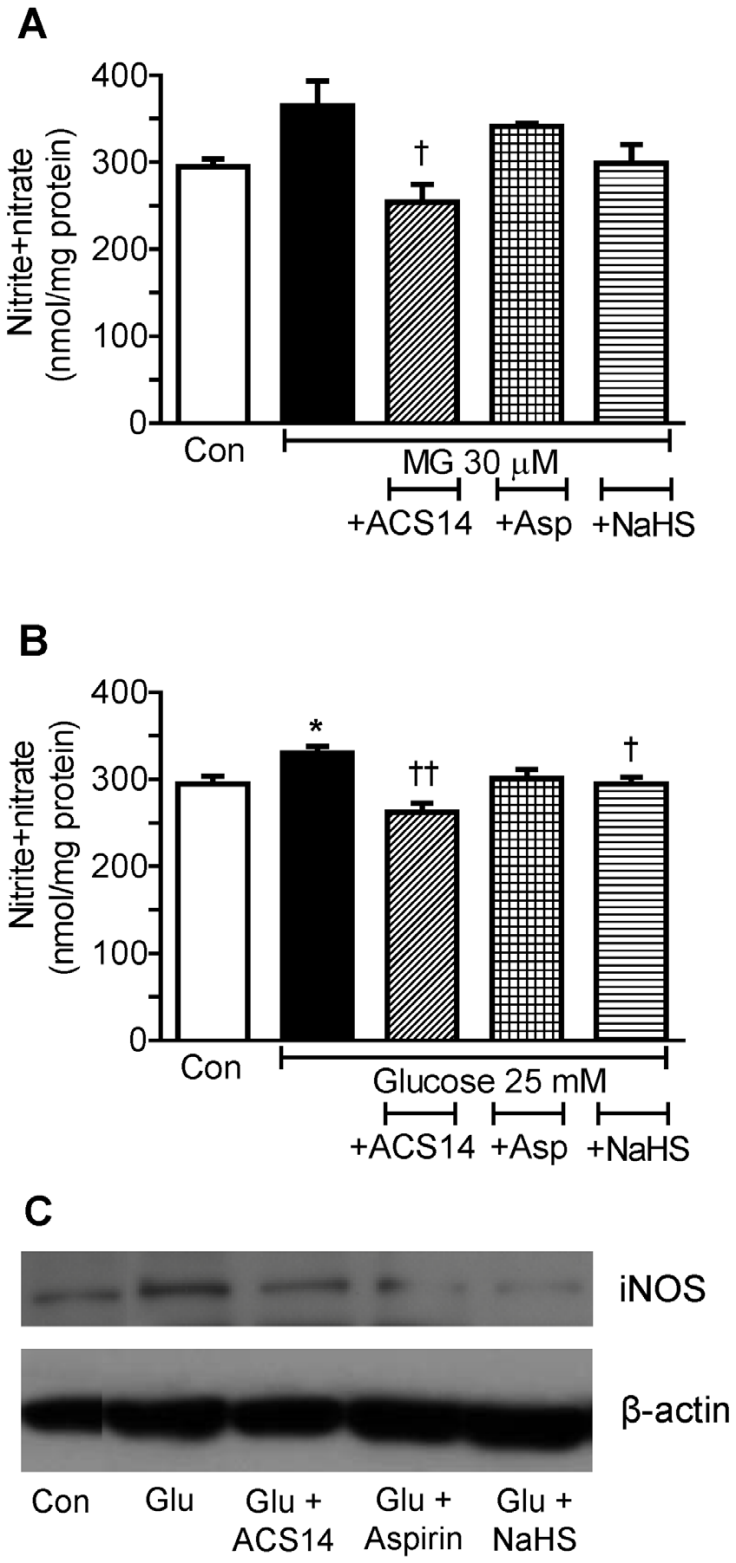
ACS14, but not aspirin, causes a significant attenuation of increase in nitrite+nitrate levels caused by MG or high glucose in cultured cells. Cultured rat aortic vascular smooth muscle cells (VSMCs, A10 cell line) were incubated with methylglyoxal (MG, 30 µM) (A), or high glucose (25 mM) (B, C), alone or co-incubated with either ACS14 (100 µM), or aspirin (100 µM) or sodium hydrogen sulfide (NaHS, 90 µM) for 24 h. Nitrite+nitrate levels in the supernatant were measured with the Griess assay kit (A, B). Expression of iNOS protein was determined with Western blotting (C). *P<0.05 *vs.* respective control, ^†^ *P*<0.05 and ^††^ *P*<0.01 *vs.* respective MG group or high glucose group.

Incubation of cultured VSMCs with high glucose (25 mM) for 24 h caused a significant elevation of nitrate+nitrite levels (Fig. 3B). Co-incubation with ACS14 significantly decreased the nitrate+nitrite levels compared to MG treated cells (Fig. 3A) and also attenuated the increase in nitrate+nitrite levels caused by 24 h incubation with high glucose (Fig. 3B). Aspirin co-treated cells did not have significantly lower levels of nitrite+nitrate compared to MG treated cells (Fig. 3A) or high glucose treated cells (Fig. 3B). NaHS co-treatment caused a significant attenuation of increase in nitrate+nitrite caused by incubation with high glucose (Fig. 3B). ACS14, aspirin and NaHS also attenuated the increase in iNOS expression caused by high glucose (25 mM) incubation for 24 h in VSMCs (Fig. 3C).

### ACS14, aspirin, and sodium hydrogen sulfide, all attenuate the increase in oxidative stress caused by MG and high glucose in cultured cells

**Figure 4:**
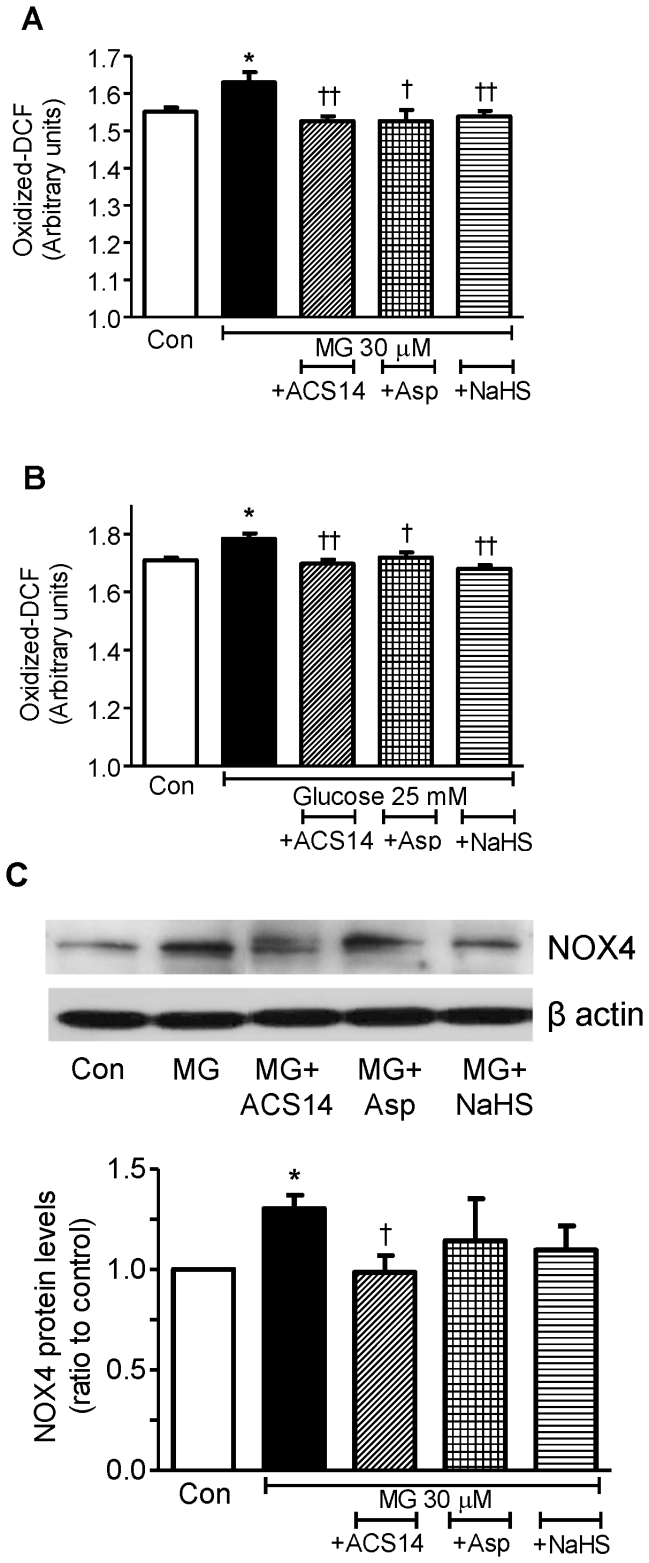
ACS14, aspirin, and sodium hydrogen sulfide, all attenuate the increase in oxidative stress caused by MG and high glucose in cultured cells. Cultured rat aortic vascular smooth muscle cells (VSMCs, A10 cell line) were incubated with methylglyoxal (MG, 30 µM) (A, C), or high glucose (25 mM) (B), alone or co-incubated with either ACS14 (100 µM), or aspirin (100 µM) or sodium hydrogen sulfide (NaHS, 90 µM) for 24 h. Oxidative stress (mainly peroxynitrite formation) was measured as oxidized dichlorofluorescein (DCF) (A, B). Western blotting was performed to measure NOX4 protein expression (C). *P<0.05 *vs.* respective control, ^†^ *P*<0.05 and ^††^ *P*<0.01 *vs.* respective MG group or high glucose group.

Incubation of cultured VSMCs with MG (30 µM) or high glucose (25 mM) for 24 h caused a significant elevation of oxidative stress measured as oxidized-DCF (Fig. 4). Co-incubation with either ACS14 (100 µM), aspirin (100 µM) or NaHS (90 µM) significantly prevented the increase in oxidative stress caused by 24 h incubation with either MG (30 µM) or high glucose (Fig. 4A, B). Since, NADPH oxidase is a major source of superoxide formation, an analysis of NOX4 protein showed significant elevation of NOX4 expression in cultured VSMCs incubated with MG (30 µM), which was attenuated by co-incubation with ACS14 (100 µM), but not with aspirin or NaHS (Fig. 4C).

### Methylglyoxal and ACS14 reduce cell viability of cultured vascular smooth muscle cells

**Figure 5:**
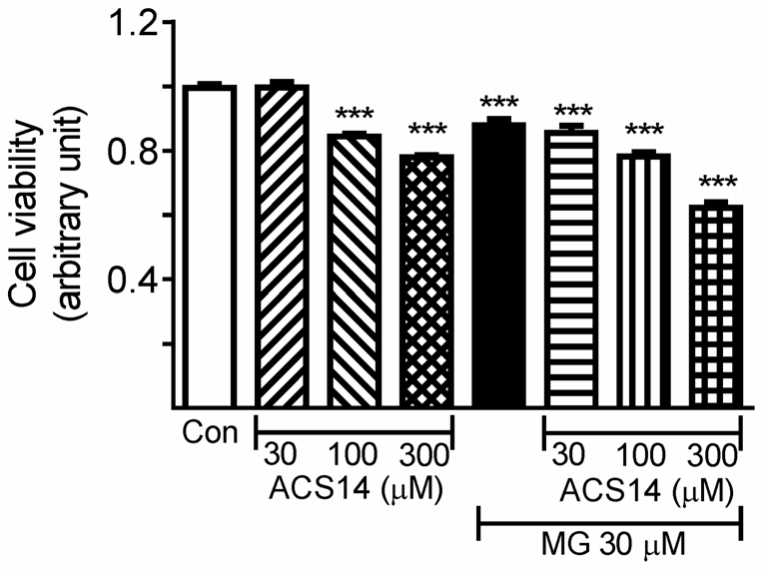
MG and ACS14 significantly reduce cell viability of cultured vascular smooth muscle cells. Cultured rat aortic vascular smooth muscle cells (VSMCs, A10 cell line) were incubated with methylglyoxal (MG, 30 µM) or ACS14 (30, 100 or 300 µM), alone or combined, for 3 h. Cell viability was determined with CellTiter 96 AQueous One Solution Cell Proliferation Assay as described in the methods. ***P<0.001 *vs.* respective control, ^†††^ *P*<0.001 *vs.* MG alone group.

Incubation of cultured VSMCs with ACS14 (30 µM) did not affect cell viability compared to control, but ACS14 (100 µM) alone caused about 15% decrease in cell viability (Fig. 5). ACS14 (300 µM) decreased cell viability by about 23%. MG (30 µM) alone decreased cell viability by about 12% (Fig. 5) whereas MG (30 µM) coincubated with ACS14 (30 µM) reduced cell viability by about 15%, and with ACS14 (100 µM) by about 20%. MG (30 µM) plus ACS14 (300 µM) reduced cell viability by about 38% (Fig. 5).

## Discussion

Here we show a novel effect of the H_2_S releasing aspirin, ACS14, to attenuate an increase in MG levels caused by treating cultured VSMCs with either exogenous MG or high glucose. ACS14 also reduced oxidative stress caused by MG or high glucose in VSMCs and also significantly reduced increased expression of NOX4 caused by MG. Moreover, ACS14 attenuated the increase in nitrite+nitrate levels caused by high glucose.

The ability of ACS14 to attenuate the increase in MG levels caused by exogenous MG or high glucose is an attractive feature of this novel drug. Endogenous glucose and fructose metabolism are the main sources of MG formation in the body [7], [16], [23], [24]. An excess of MG formation in the body as seen in diabetic patients [14], [15] and rats fed a high fructose diet [23], [25] is harmful and can cause pathologies such as endothelial dysfunction and features of type 2 diabetes [8], [17]. Moreover, MG is a major precursor for the formation of AGEs [10]. The reaction of MG with arginine produces hydroimidazolones such as Nε-(5-hydro-5-methyl-4-imidazolon-2-yl)-ornithine and argpyrimidine [26], whereas with lysine it forms Nε-carboxyethyllysine CEL [27]. Thus, ACS14 has the potential to prevent the harmful effects of elevated MG and also provide antithrombosis [28] in diabetic patients, who have an increased risk of developing cardiovascular complications. We have previously shown that H_2_S provided by NaHS decreases MG levels in VSMCs [18].

ACS14 also reduced oxidative stress. We are using the term “oxidative stress” because the probe 2′,7′-dichlorofluorescein diacetate (CM-H2DCFDA) is not absolutely specific for peroxynitrite even though it has high specificity for peroxynitrite and low for hydrogen peroxide and superoxide [21]. ACS14 has been shown to reduce oxidative stress in other studies [5], [6]. MG is a major trigger for increasing oxidative stress [29], [30] and since ACS14 prevents an increase in MG levels, this could be one of the mechanisms by which ACS14 reduces oxidative stress besides causing an increase in the antioxidant GSH levels [6]. We have previously shown that MG and high glucose can increase oxidative stress [8], [16], [29], [31], which can be attributed to increased activity of NADPH oxidase [8] [8]and NF-κB [29]. We have also shown that MG and high glucose can increase the expression of NF-κB and NOX4 protein in cultured VSMCs and human umbilical vein endothelial cells [31]. MG is a potent inducer of oxidative stress as discussed in a review by us [30], and scavenging MG would prevent activation of multiple pathways of increased free radical generation. Thus, incubation of cultured VSMCs with 30 µM MG for 24 h increased the expression of NOX4, which was attenuated by co-incubation with ACS14. The reduced expression of NOX4 caused by ACS14 in the current study could be due to an attenuation of MG levels in VSMCs. NOX4 is a potential source of superoxide and increased oxidative stress in VSMCs [32], [33].

ACS14, but not aspirin, attenuated an increase in nitrite+nitrate levels caused by high glucose. High glucose caused increased expression of iNOS which was attenuated by ACS14 (Fig. 3C). We have previously shown that MG caused an increase in nitrite+nitrate levels in VSMCs, most probably coming from increased expression of inducible nitric oxide synthase (iNOS) [16]. Increased nitric oxide production from iNOS can potentially react with superoxide and cause increased peroxynitrite formation detected as oxidized dichlorofluorescein in the current study.

ACS14 100 µM caused about 15% decrease in cell viability whereas 30 µM of ACS14 did not. Thus, about 85% of cells survived at ACS14 100 µM (*vs.* control). ACS14 at 100 µM produced more consistent attenuation of the effects of MG and since cell viability decreased by only about 15% at that concentration we decided to use 100 µM of ACS14. The results of cell viability also caution us not to use ACS14 beyond a certain concentration or dose due to increased cytotoxicity with higher concentrations. This makes sense because H_2_S has been shown to be toxic at higher concentrations.

Limitations of the study. Besides NOX4 we have previously shown that MG and high glucose increase the expression of NF-κB in cultured VSMCs [29], [31]. Thus, it would have been useful to examine the effect of MG and ACS14 on NF-κB expression. Similarly, it would have been useful to measure levels of reduced and oxidized glutathione since high glucose and MG have been shown to reduce levels of reduced glutathione (GSH) and expression of glutathione reductase in cultured human umbilical vein endothelial cells [8]. Although NOX1 and NOX4 are expressed in rat VSMCs, they have different subcellular locations and functions [33]. For example one study has shown that NOX1 mediated angiotensin II induced superoxide production in rat VSMCs with a four-fold increase in NOX1 mRNA after 8 h and a 40% decrease in NOX4 mRNA [34]. Thus, it is possible that different isoforms respond to different ligands and they might even be antagonistic to each other. For example, in VSMCs from the aortas of mice after incubation with high glucose (25 mM) for 24 h, NOX4 expression increased by 250±30% whereas NOX1 increased by only 70±9% [32]. Since in our previous study NOX4 expression increased after high glucose (25 mM) and MG (30 µM) [31], we examined the effect of ACS14 on NOX4 expression. However, it would be interesting to examine the effect of MG on NOX1 expression.

A strong link between oxidative stress and inflammation has been reported previously [35], [36]. Our lab has also previously shown that incubation of neutrophils with MG (20 µM) for 12 h increases secretion of tumor necrosis factor-α (TNF-α), interleukin-6 (IL-6) and interleukin-8 (IL-8) [14]. Thus, it would have been useful to examine markers of inflammation, but aspirin is well established as an anti-inflammatory drug. Moreover, the anti-inflammatory effect of ACS14 has been previously demonstrated in cultured microglial cells [37].

In conclusion, ACS14 has the novel ability to attenuate an increase in MG levels which in turn can reduce oxidative stress, decrease AGEs formation and prevent many of the known deleterious effects of elevated MG. Thus, ACS14 has the potential to be especially beneficial for diabetic patients for which further *in vivo* studies are required.
